# Blockade but Not Overexpression of the Junctional Adhesion Molecule C Influences Virus-Induced Type 1 Diabetes in Mice

**DOI:** 10.1371/journal.pone.0054675

**Published:** 2013-01-25

**Authors:** Selina Christen, Ken Coppieters, Kerstin Rose, Martin Holdener, Monika Bayer, Josef M. Pfeilschifter, Edith Hintermann, Matthias G. von Herrath, Michel Aurrand-Lions, Beat A. Imhof, Urs Christen

**Affiliations:** 1 Pharmazentrum Frankfurt/ZAFES, Goethe University Hospital, Frankfurt am Main, Germany; 2 The La Jolla Institute for Allergy and Immunology, La Jolla, California, United States of America; 3 Inserm, Centre de Recherche en Cancérologie de Marseille, Inserm, CNRS, Aix-Marseille Université, UMR 891, Marseille, France; 4 Department of Pathology and Immunology, Centre Médical Universitaire, Geneva, Switzerland; University of Bremen, Germany

## Abstract

Type 1 diabetes (T1D) results from the autoimmune destruction of insulin-producing beta-cells in the pancreas. Recruitment of inflammatory cells is prerequisite to beta-cell-injury. The junctional adhesion molecule (JAM) family proteins JAM-B and JAM–C are involved in polarized leukocyte transendothelial migration and are expressed by vascular endothelial cells of peripheral tissue and high endothelial venules in lympoid organs. Blocking of JAM-C efficiently attenuated cerulean-induced pancreatitis, rheumatoid arthritis or inflammation induced by ischemia and reperfusion in mice. In order to investigate the influence of JAM-C on trafficking and transmigration of antigen-specific, autoaggressive T-cells, we used transgenic mice that express a protein of the lymphocytic choriomeningitis virus (LCMV) as a target autoantigen in the β-cells of the islets of Langerhans under the rat insulin promoter (RIP). Such RIP-LCMV mice turn diabetic after infection with LCMV. We found that upon LCMV-infection JAM-C protein was upregulated around the islets in RIP-LCMV mice. JAM-C expression correlated with islet infiltration and functional beta-cell impairment. Blockade with a neutralizing anti-JAM-C antibody reduced the T1D incidence. However, JAM-C overexpression on endothelial cells did not accelerate diabetes in the RIP-LCMV model. In summary, our data suggest that JAM-C might be involved in the final steps of trafficking and transmigration of antigen-specific autoaggressive T-cells to the islets of Langerhans.

## Introduction

The pathogenesis of T1D is characterized by the destruction of insulin producing β-cells by autoaggressive lymphocytes invading the islets of Langerhans. This inflammatory processes can be driven by infection with a pancreas-tropic virus or toxin-induced β-cell necrosis, resulting in the attraction of autoaggressive T cells to the islets of Langerhans. Local expression of chemokines and subsequently the upregulation of a variety of adhesion molecules by endothelial cells facilitate the attraction and transmigration of leukocytes from the circulation to the islets. We have demonstrated in the past that blockade of critical chemokines, such as CXCL10 (IP-10, IFNγ-inducible protein of 10 kDa), results in the abrogation of T1D in the RIP-LCMV model [1] indicating that cellular attraction to the islet of Langerhans is a critical step required for the subsequent destruction of insulin-producing β-cells.

Besides chemokine-mediated attraction of leukocytes to the site of inflammation, extravasation from the blood vessels through the endothelial cell layer is required for penetration into the islets. Within the leukocyte-extravasation cascade, selectins initiate leukocyte tethering and rolling and the interaction between integrins and immunoglobulins is required for firm adhesion and transmigration [2], [3]. Selectin-induced rolling allows for a close proximity to endothelial cells and binding of chemokines (such as CXCL10) that are displayed on inflamed endothelium. Subsequently, leukocytes are activated via their chemokine receptors and an array of integrins is expressed at the leukocyte surface. Interactions between β2-integrin and intracellular adhesion molecule-1 (ICAM-1) as well as very late antigen-4 (VLA-4) and vascular cell adhesion molecule-1 (VCAM-1) are crucial for firm adhesion of leukocytes to the inflamed endothelium [2], [3]. Finally, interaction between JAM-C, which is predominantly expressed on endothelial cells and the β2-integrin CD11b present on leukocytes, including diabetogenic T cells in T1D, is required for the transmigration from the lumen through the endothelial cell layer into the inflamed tissue [2], [3]. ICAM-1 seems to be a key adhesion molecule during the T1D pathogenesis, since ICAM-1-deficient NOD mice are protected from T1D and cellular islet infiltration was strongly reduced when compared to age-matched regular NOD mice [4]. In the RIP-LCMV model for T1D ICAM-1 is upreguated around the islets of Langerhans upon LCMV-infection [5]. In addition, blockade of ICAM-1 resulted in a reduced infiltration of diabetogenic T cells into the islets of RIP-HEL mice, that express hen-egg white lysozyme (HEL) in the β-cells [6]. Interestingly, blockade platelet endothelial cell adhesion molecule-1 (PECAM-1) had no effect on T cell infiltration although it was strongly expressed on islet vessels [6]. Mice lacking ICAM-1 are partially protected from cerulein-induced pancreatitis [7], but the administration of anti-ICAM-1 antibodies had only little effect [8].

In contrast to ICAM-1, blockade of JAM-C with a neutralizing antibody reduced the severity of cerulein-induced pancreatitis and overexpression of JAM-C on endothelial cells enhanced the cellular infiltration and the acinar cell necrosis [9]. In contrast to T1D, severe pancreatitis predominantly affects the exocrine part of the pancreas resulting in the necrosis of acinar cells [8], [9]. Thus, we intended to further investigate if JAM-C is also important in pathogenesis of T1D in the virus-induced RIP-LCMV model. The RIP-LCMV model uses either the nucleoprotein (NP) or the glycoprotein (GP) of LCMV as target antigens expressed by the β-cells. T1D is induced at a defined time by infection with LCMV [10]. Thus, the RIP-LCMV allows for a precise characterization of the inflammation kinetics occurring in the islets of Langerhans after induction of the autodestructive processes [11]. In the present work, we analyzed the expression of JAM-C after LCMV-infection and applied an anti-JAM-C therapy for T1D using neutralizing antibodies. Further, we assessed if overexpression of JAM-C in endothelial cells accelerates T1D pathogenesis.

## Materials and Methods

### Mice and Virus

Generation and screening by PCR of H-2^b^ RIP-LCMV-GP and H-2^b^ RIP-LCMV-NP transgenic mice were as previously described [10], [12]. pHHNS-JAM-C transgenic mice have been generated as describe elsewhere [13] and have been backcrossed to the C57BL/6 background for more than ten generations. pHHNS-JAM-C mice have been crossed to RIP-LCMV-GP and RIP-LCMV-NP mice and genotyped by PCR as previously described for JAM-C [13] and LCMV-GP and –NP [10], [12]. LCMV Armstrong clone 53 b (LCMV-Arm) were plaque-purified three times on Vero cells and stocks were prepared by a single passage on BHK-21 cells [12]. For induction of T1D in RIP-LCMV-GP, RIP-LCMV-NP, as well as the double transgenic RIP-LCMV lines crossed to pHHNS-JAM-C mice 10^4^ plaque forming units (pfu) LCMV-Arm were injected intraperitoneally. All animal experiments have been approved by the local Ethics Animal Review Board, Darmstadt, Germany (Reference number: V54-19c20/15-F143/30).

### Blood Glucose Values

Blood samples were obtained from the tail vein. Blood glucose was monitored with a OneTouch Ultra at weekly intervals. Animals with blood glucose values over 300 mg/dl were considered diabetic [5].

### Anti-JAM-C Antibody

Rat-anti mouse JAM-C monoclonal antibody clone H33 ([14] was purified from rat hybridoma cells grown in DMEM, 1% Ultroser HY and 0.1% FBS. Briefly, the collected hybridoma supernatants were filtered, precipitated with 45% of ammonium sulphate, resuspended in PBS and dialysed against PBS. Then the anti-JAM-C antibodies were purified over Protein G sepharose 4 fast flow over night at 4°C on an end-over-end rotation. Thereafter the sepharose beads were washed with PBS, the antibodies were eluted with 0.1 M Triethanolamine (pH 10.5) and immediately neutralized with 2 M Tris pH 7.5. For *in vivo* blockade experiments a rat IgG2a antibody to human CD44 (9B5) [9] was used a isotype-matched control antibody.

### Immunohistochemistry

Tissues were immersed in Tissue-Tek OCT (Bayer), and quick-frozen on dry ice. Using cryomicrotome and sialin-coated Superfrost Plus slides (Fisher Scientific), 6 µm tissue sections were cut. Sections were then fixed with 90% ethanol at −20°C, and, after washing in PBS, an avidin/biotin-blocking step was included (Vector Laboratories). Primary and biotinylated secondary antibodies (Vector Laboratories) were reacted with the sections for 60 min each, and color reaction was obtained by sequential incubation with avidin-peroxidase conjugate (Vector Laboratories) and diaminobenzidine-hydrogen peroxide. Primary antibodies used were: rat anti-mouse CD8a, rat anti-mouse CD4, rat anti-mouse CD31 (all from BD Biosciences, Heidelberg, Germany) and a polyclonal rabbit anti-JAM-C antibody described elsewhere [9].

### Immunofluorescence Staining

Pancreas tissue sections of 6 µm were fixed in 90% ethanol at −20°C, washed in PBS and blocked in PBS containing 10% FBS for 30 min. Primary antibodies rat anti-mouse CD8a (5 µg/ml), rat anti-mouse CD31 (2.5 µg/ml) (both from BD Biosciences, Heidelberg, Germany) and a polyclonal rabbit anti-JAM-C antibody (5 µg/ml) described elsewhere [9] were incubated for 2 h at room temperature in a humified chamber. An Alexa 488-conjugated goat anti-rabbit IgG (5 µg/ml) and a Texas Red-conjugated goat anti-rat IgG (2.5 µg/ml) were used as secondary antibody. Section were counterstained with 4,6-diamidino-2-phenylindiole (DAPI) (200 ng/ml) (Sigma-Aldridge, Munich, Germany). Fluorescence images were acquired with an Axiovert 135 TV fluorescence microscope (Zeiss, Jena, Germany). Fluorescence intensities were determined using the Quantity One software (Bio Rad, Munich, Germany).

### Flow Cytometry

For intracellular stains, single-cell suspensions of spleens and PDLNs were restimulated overnight with MHC class I- or class II-restricted viral peptides (2 µg/ml LCMV-GP_33_, LCMV-NP_396_, or LCMV-GP_61_) in the presence of Brefeldin A. Cells were stained for surface expression of CD8 and CD4 and fixed, permeabilized and stained for intracellular IFNγ as previously described [5]. All antibodies were obtained from BD-Biosciences, Heidelberg, Germany. Samples were acquired a FACS Canto II flow cytometer (BD-Biosciences, Heidelberg, Germany).

### Determination of Viral Titers by Plaque Assay

Viral titers of organ homogenates were determined by infection of Vero cells as described [12], [15]. Tissues (spleen and pancreas) were obtained from RIP-LCMV-GP and RIP-LC**MV-**GP × pHHNS-JAM-C (three animals per group) at days 3 and 7 after infection with 1×10^4^ LCMV (i.p.). Homogenates and sera were diluted serially, and viral titers were calculated from the number of counted plaques.

### Statistical Evaluations

Diabetes incidence curves (‘survival curves’) were analyzed using the Log-rank (Mantel-Cox) test (GraphPad Prism 5.02 Software). Contingency of islet infiltration/insulitis score was analyzed by the chi-square test and T-cell frequencies were analyzed using the non-parametric, unpaired, two-tailed Mann-Whitney test.

## Results

### LCMV-infection Results in an Upregulation of JAM-C Around the Islets of Langerhans

**Figure 1:**
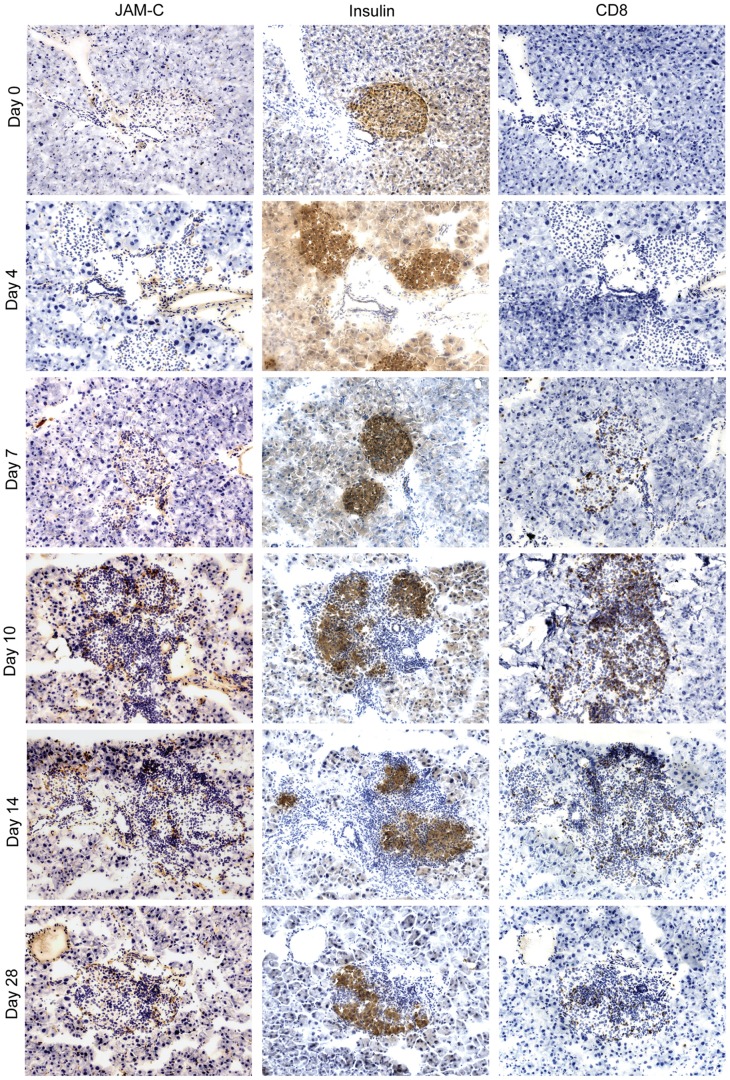
JAM-C expression correlates with islet infiltration and functional impairment. - Pancreas sections of RIP-LCMV-GP mice were probed for JAM-C expression, insulin production and CD8^+^ T cell infiltrations at different times after LCMV-infection.

**Figure 2:**
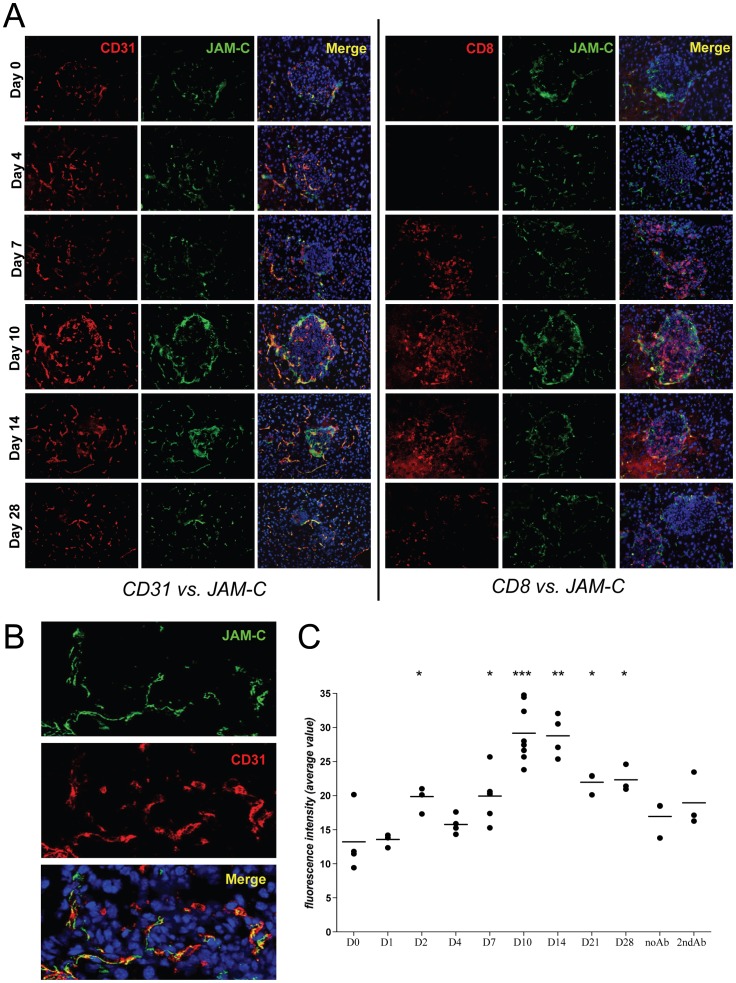
Expression of JAM-C after LCMV-infection. – (A) Pancreas sections of RIP-LCMV-GP mice were analyzed for expression of CD31 (red) and JAM-C (green) as well as CD8^+^ T cell infiltrations (red) and JAM-C (green) at different times after LCMV-infection. (B) JAM-C expression (green) predominantly co-localized with the endothelial cell marker CD31 (red) as shown in higher magnification in a representative pancreas section from a RIP-LCMV-GP mouse at day 10 post-infection. (C) Evaluation of fluorescence intensity of JAM-C expression at different times after LCMV-infection. Bars representing mean of 3–8 islets each time point and statistical significance of the fluorescence intensity relative to day 0 were determined using the unpaired t test (*, p<0.05; **, p<0.005; ***, p<0.0005).

To assess the kinetics of JAM-C expression pancreata from RIP-LCMV-GP mice were harvested at day 0, 4, 7, 10, 14, and 28 after i.p. infection with 10^4 ^pfu LCMV and analyzed by immunohistochemistry or immunofluorescence staining. JAM-C was upregulated around the islets predominantly at days 10 and 14 after LCMV-infection (Figure 1, left panel and Figure 2A). Interestingly, cellular islet infiltration was most pronounced at the time of strongest JAM-C expression (Figure 1 and Figure 2A, right panel). At day 10 to 14 post-infection most islets of LCMV-infected RIP-LCMV-GP mice showed massive peri- and intra-islet CD8^+^ T cell infiltrations (Figure 1, right panel and Figure 2A, right panel). The presence of high numbers of T cells in and around the islets resulted in the destruction of islet cells and impaired β-cell function that finally led to overt disease (Figure 1, middle panel). For immunofluorescence stainings the constitutively expressed adhesion molecule, CD31, was used as an endothelial cell marker and the co-localization of JAM-C and CD31 was characterized by confocal microscopy (Figure 2B). JAM-C staining co-localized with CD31 on endothelial cells from pancreatic blood vessels (Figure 2A and B). As seen previously (Figure 1) a significant increase in fluorescence intensity of JAM-C expression was detected at day 10 to 14 after LCMV-infection (Figure 2C). Quantification of the mean intensity of the individual fluorescence channels revealed the highest JAM-C expression at days 10 and 14 post-infection (Figure 2C). In comparison to the pancreas of uninfected RIP-LCMV-GP mice, the expression of JAM-C was increased significantly (p<0.05) at days 2, 7, peaked at day 10 (p = 0.0001) and day 14 (p = 0.015), and declined again at day 21 and 28 after LCMV-infection (Figure 2C). The quantification of the immunofluorescence staining confirmed the observation that JAM-C expression parallels the peak of CD8 T cell infiltration (Figure 1 and 2A).

### JAM-C Blockade: Neutralization of JAM-C Results in a Decreased T1D Incidence

**Figure 3:**
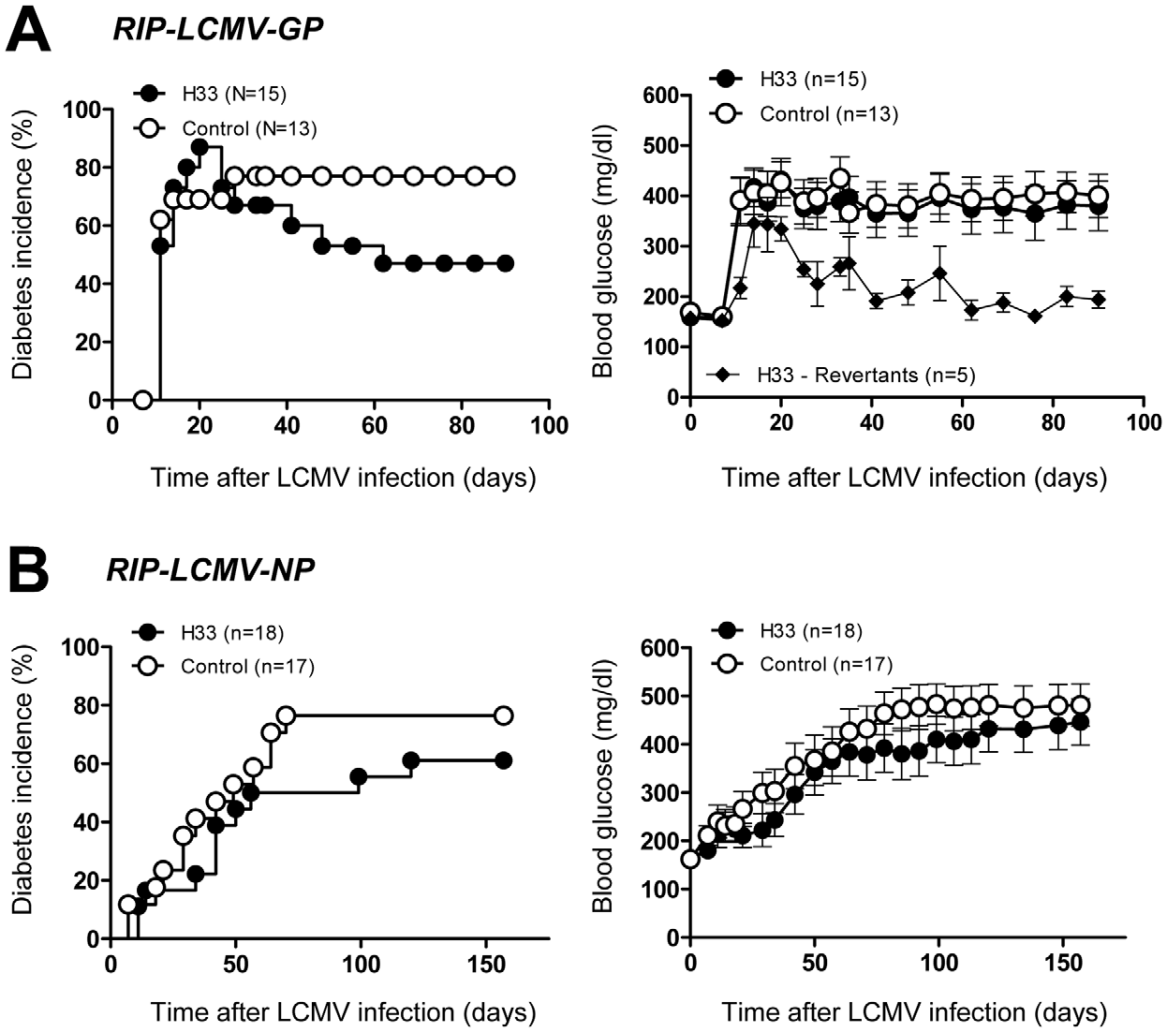
Neutralization of JAM-C reduces the development of T1D. - RIP-LCMV-GP (A) and RIP-LCMV-NP (B) mice were infected with LCMV. Mice were treated thrice weekly with 100 µg of anti-JAM-C mAb (H33) (filled circles) or isotype-matched control antibody (open circles). Blood glucose was measured at weekly intervals. For the determination of the incidence mice with values >300 mg/dl were considered diabetic (left panels). Blood glucose data of all mice are displayed as mean ± SEM of 13–18 mice per group (right panels). In addition, the mean blood glucose levels of diabetic RIP-LCMV-NP mice showing a reversion to a non-diabetic state after treatment with anti-JAM-C mAb (reverants) are presented separately (filled diamonds).

In order to investigate if JAM-C might also play a role in the actual destruction of β-cells and the immunopathogenesis of T1D, we blocked JAM-C with a neutralizing anti-JAM-C antibody that disrupts murine JAM-B/−C interactions [16]. In RIP-LCMV-GP mice anti-JAM-C antibody (100 µg each injection) was administered i.p. one day before 10^4 ^pfu LCMV-infection and then at days 1, 2, 5, 8, 11, and 14 post-infection. Anti-JAM-C antibody-treated as well as control RIP-LCMV-GP mice displayed a similar initial diabetes incidence of approximately 80% within the first 21 days after LCMV-infection (Figure 3A). Interestingly, neutralization of JAM-C led to a reversion of T1D two months after LCMV-infection in that only 47% of the anti-JAM-C antibody-treated mice remained diabetic compared to 77% of diabetic control animals. Reverting mice remained non-diabetic throughout the whole observation period (Figure 3A). In RIP-LCMV-NP mice anti-JAM-C antibody (100 µg each injection) was administered i.p. one day before 1×10^4 ^pfu LCMV-infection and then at days 1, 3, 7, post-infection followed by injections twice weekly until day 34 post-infection. In the RIP-LCMV-NP mice the incidence of diabetes in JAM-C antibody-treated mice was slightly delayed and reduced to 61% compared to an incidence of 76% in control mice (Figure 3B). However, this reduction was not significant as revealed by a log rank (Mantel-Cox) analysis of the survival curves. The observed minor reduction in T1D severity in RIP-LCMV-GP and RIP-LCMV-NP mice was only marginally reflected in the mean blood glucose values. Treatment with anti-JAM-C antibody resulted in a non-significant decrease of mean blood glucose levels in RIP-LCMV-NP mice (Figure 3B, right panel). In contrast, the observed reversion of T1D in some RIP-LCMV-GP mice was not obvious when analyzing the mean blood glucose levels of all mice (Figure 3A, right panel). However, the mean blood glucose decreased markedly when considering the revertant mice only (Figure 3A, right panel). The rather high variation (SEM) in blood glucose values results from the different T1D onset in individual mice of the same group, with some diabetic mice with blood glucose levels of up to 600 mg/dl and some still non-diabetic mice with levels below 200 mg/dl.

**Figure 4:**
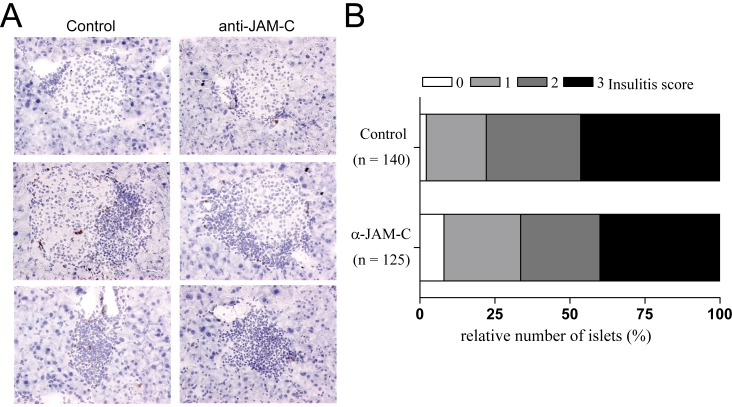
Neutralization of JAM-C has no influence on insulitis. – (A) Pancreas sections of RIP-LCMV-NP mice were stained for CD8 T cells at months 6 after LCMV-infection (study end point). (B) Insulitis score was determined in pancreas sections of 8–9 RIP-LCMV-NP mice per group (n = 140, islets analyzed). Scoring system: 0, no infiltration; 1, some peri-insular infiltration; 2, heavy peri-insular infiltration with some intra-insular infiltrates; 3, heavy intra-insular infiltration and/or islet scars. Representative sections for score 1 to 3 are displayed for control and anti-JAM-C antibody treated mice (A).

JAM-C blockade had no effect on viral clearance of LCMV, since similar viral titers have been found in the pancreas and the spleen at days 3, 7 and 14 after infection (data not shown). We then compared islet infiltration in RIP-LCMV-NP mice treated with JAM-C neutralizing antibody or isotype control at the end point of the study at six months after LCMV-infection (Figure 4). For semi-quantitative assessment of the islet infiltration, an infiltration score was applied and statistical evaluation was performed after blinded analysis of the tissue sections. No significant difference in islet infiltration was found in anti-JAM-C antibody-treated mice compared to control animals (Figure 4A+B). Nevertheless, the blocking studies in RIP-LCMV mice suggest that JAM-C plays a certain role in the pathogenesis of T1D, since neutralization of JAM-C resulted in a reversion of T1D in some diabetic RIP-LCMV-GP mice (fast-onset model) and a reduction of mean blood glucose levels and overall T1D incidence RIP-LCMV-NP mice (slow-onset model).

### JAM-C Blockade: The Frequency of LCMV-specific T Cells is not Significantly Reduced After Neutralization of JAM-C

**Figure 5:**
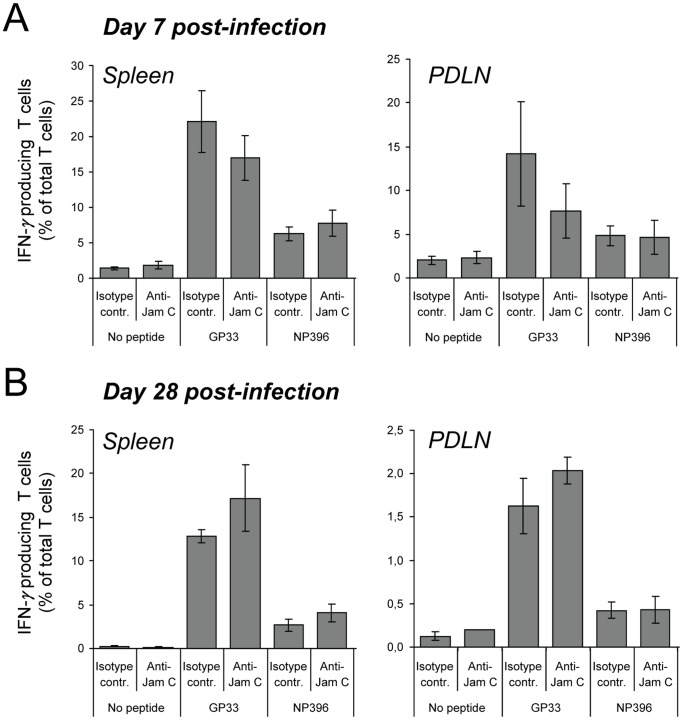
Neutralization of JAM-C has no significant influence on the frequency of LCMV-specific T cells. - The presence of LCMV-specific CD8 T cells on day 7 (A) and 28 (B) after LCMV-infection in the spleen and PDLN of RIP-LCMV-NP mice treated with anti-JAM-C antibody or isotype-matched control antibody was analyzed by ICCS (IFN-γ) after o/n *in vitro* stimulation with LCMV-GP_33_ or LCMV-NP_396_ peptide. Data are the mean ± SD of 3–4 mice per group. As internal controls unstimulated cells as well as lymphocytes from a wildtype C57BL/6 mouse (wt) were used.

We next assessed the functional activity of LCMV-specific T cells after JAM-C blockade in RIP-LCMV-NP mice. Anti-JAM-C antibody or an isotype-matched control antibody (100 µg each injection) was administered i.p. one day before 1×10^4 ^pfu LCMV-infection and then at days 1, 3, 7, post-infection followed by injections twice weekly until day 26 post-infection. At days 7 and 28 post-infection lymphocytes were isolated from the spleen and the PDLN and were stained for CD8 and IFN-γ after overnight stimulation with the immunodominant LCMV-peptides NP_396_ and GP_33_. At day 7 post-infection a tendency towards a reduction of GP_33_-specific CD8 T cells was observed in the spleen and particularly in the PDLN of anti-JAM-C antibody-treated mice compared to control animals (Figure 5A). In contrast, during the autoimmune phase at day 28 post-infection the frequency of GP_33_-specific CD8 T cells was similar in JAM-C blocked and control mice (Figure 5B). More important for the evaluation of the autodestructive process in RIP-LCMV-NP mice is the frequency of NP_396_-specific CD8 T cells. However, both at days 7 and 28 the frequency of such diabetogenic CD8 T cells was similar in the spleen and the PDLN of mice that have been treated with the anti-JAM-C antibody and isotype-matched control antibody (Figure 5). The results indicate that blockade of JAM-C might have a minor effect on the initial expansion of LCMV-specific CD8 T cells after LCMV-infection, but does not influence the frequency of islet-antigen (LCMV-NP)-specific CD8 T cells during the autoimmune process β-cell destruction.

### Virus-induced Diabetes is not Accelerated in Mice Expressing Transgenic JAM-C on Pancreatic Endothelial Cells

**Figure 6:**
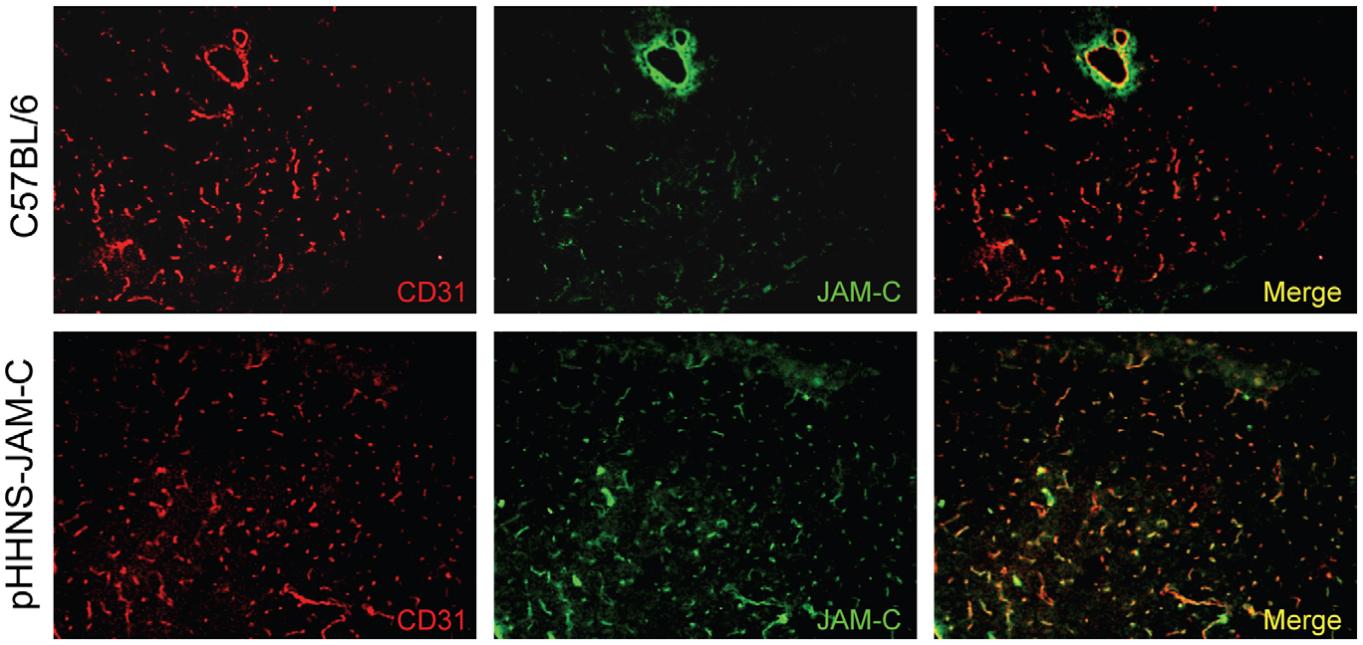
Characterization of JAM-C expression in the pancreas of pHHNS-JAM-C transgenic mice. - Immmunofluorescence staining of pancreas sections of pHHNS-JAM-C transgenic and wildtype C57BL/6 mice for JAM-C and the endotheial cell marker CD31. Note that the expression of transgenic JAM-C co-localizes with CD31 expressing endothelial cells.

In order to further investigate a possible influence of JAM-C on the immunopathogenesis of T1D, we infected pHHNS-JAM-C mice that express JAM-C under control of the tie2-promotor specifically on endothelial cells [13]. Pancreas sections from pHHNS-JAM-C transgenic and wildtype mice were analyzed for expression of JAM-C on endothelial cells by immunofluorescence co-staining with the endothelial cell marker CD31. In comparison to wildtype C57Bl/6 mice, pHHNS-JAM-C mice express increased levels of JAM-C on pancreatic blood vessels throughout the entire pancreas section (Figure 6).

**Figure 7:**
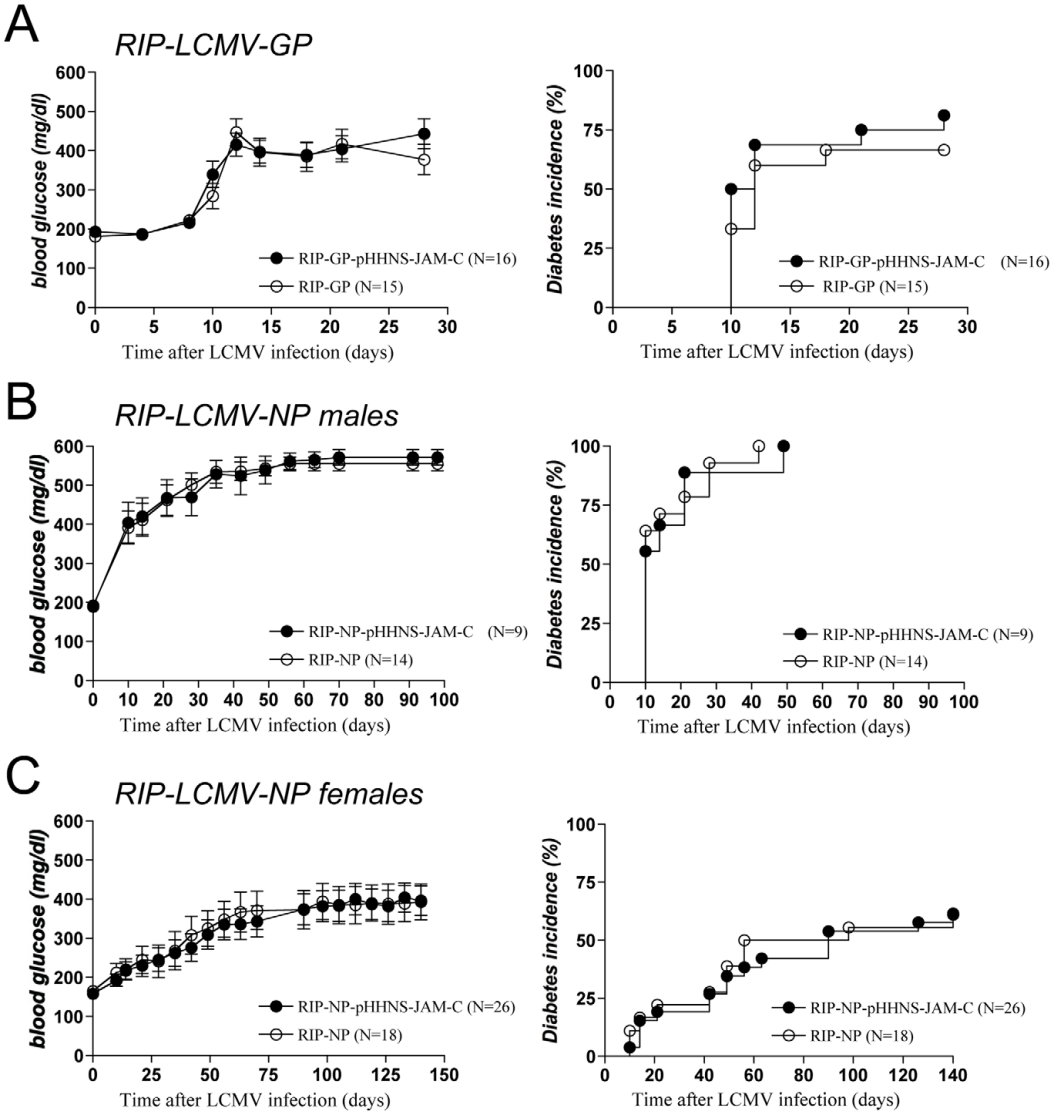
JAM-C overexpression does not influence the outcome of T1D. - Mean blood glucose levels (left panels) and diabetes incidence (right panels) of single transgenic RIP-LCMV and double transgenic RIP-LCMV × pHHNS–JAM-C mice that were infected with a single dose of LCMV. Blood glucose was measured at weekly intervals (values >300 mg/dl were considered diabetic). Data are the mean ± SEM of 9–26 mice per group. (A) RIP-LCMV-GP × pHHNS–JAM-C transgenic mice. (B) Male RIP-LCMV-NP × pHHNS–JAM-C transgenic mice. (C) Female RIP-LCMV-NP × pHHNS–JAM-C transgenic mice.

We crossed pHHNS-JAM-C mice to RIP-LCMV-GP and RIP-LCMV-NP mice to evaluate if JAM-C overexpression might result in an acceleration of T1D. Similar to single transgenic RIP-LCMV-GP littermates, double transgenic RIP-LCMV-GP × pHHNS-JAM-C mice developed T1D in a highly synchronized fashion between days 12 to 15 (Figure 7A). We found a gender difference in the T1D incidence in LCMV-infected RIP-LCMV-NP × pHHNS-JAM-C mice (Figure 7B and 7C). Whereas male RIP-LCMV-NP and RIP-LCMV-NP × pHHNS-JAM-C mice followed a similar pattern of blood glucose elevation as RIP-LCMV-GP mice (Figure 7B), female RIP-LCMV-NP and RIP-LCMV-NP × pHHNS-JAM-C mice developed T1D within 2 to 5 months post-infection (Figure 7C). However, both the kinetics and overall incidence of T1D was similar in RIP-LCMV-NP and RIP-LCMV-NP × pHHNS-JAM-C mice (Figure 7C). In summary, we observed that overexpression of JAM-C on endothelial cells did not have any effect on LCMV-induced T1D neither in the fast-onset RIP-LCMV-GP line nor in the slow-onset RIP-LCMV-NP line.

### Viral Clearance, Islet Infiltration, and Frequency of LCMV-specific T Cells are not Changed in RIP-LCMV Mice Expressing Transgenic JAM-C on Pancreatic Endothelial Cells

**Figure 8:**
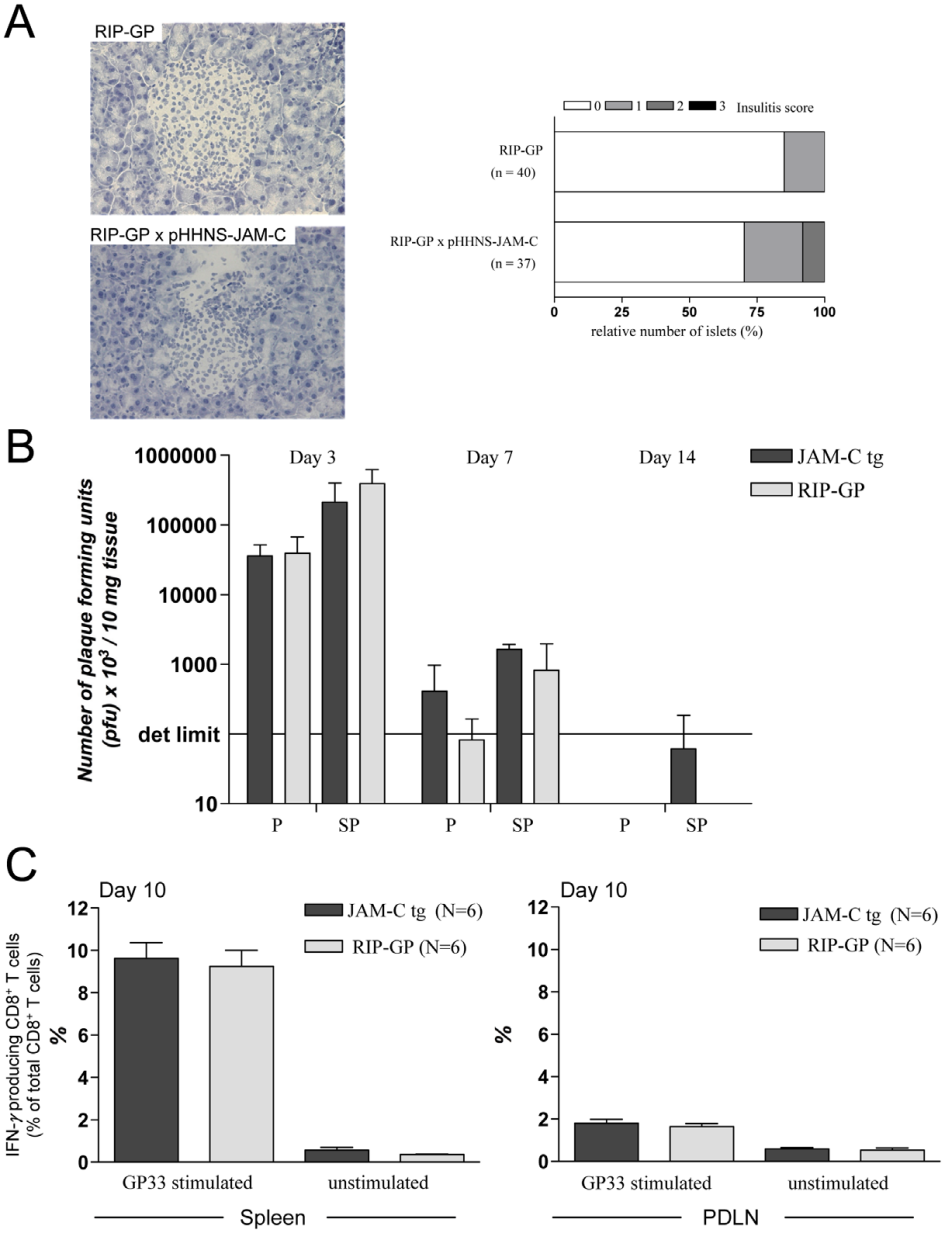
Increased JAM-C expression has no influence on islet infiltration, viral clearance or antigen specific immune response. - (A) Pancreas sections of RIP-LCMV-GP × pHHNS–JAM-C transgenic mice were stained with Hämatoxylin and the insulitis score was obtained from sections of 3 mice per group (number of islets analyzed per group are indicated). Scoring system: 0, no infiltration; 1, some peri-insular infiltration; 2, heavy peri-insular infiltration with some intra-insular infiltrates; 3, heavy intra-insular infiltration and/or islet scars. (B) Virus titers in the pancreas (P) and spleen (SP) on days 3, 7 and 14 after infection. Data are the mean ± SD of 3–4 mice per group. Det limit: Minimal number of plaques at highest tissue homogenate concentration required for statistical evaluation. (C) The presence of GP_33_-specific CD8^+^ T cells on day 10 after LCMV-infection in the spleen and PDLN of RIP-LCMV-GP or RIP-LCMV-GP × pHHNS-JAM-C mice was analyzed by intracellular cytokine staining (IFN-γ) after o/n *in vitro* stimulation with GP_33_ peptide. Data are the mean ± SD of six mice per group.

In order to confirm that increased JAM-C expression has only a minor effect on the immunopathology of virus-induced diabetes we first assessed the cellular infiltration into the islets of Langerhans in RIP-LCMV-GP × pHHNS-JAM-C mice. RIP-LCMV-GP and RIP-LCMV-GP × pHHNS-JAM-C mice were infected with 1×10^4 ^pfu LCMV and the pancreata were removed at day 6 post-infection. We have chosen such an early time in order to evaluate a possible acceleration of islet infiltration in JAM-C overexpressing mice compared to regular RIP-LCMV-GP mice. However, we were not able to detect a significant increase in insulitis at day 6 post-infection (Figure 8A). At days 10 and 14 post-infection the islets of RIP-LCMV-GP and RIP-LCMV-GP × pHHNS-JAM-C mice were already fully infiltrated (data not shown).

Second, spleens and pancreata from RIP-LCMV-GP × pHHNS-JAM-C and RIP-LCMV-GP mice were harvested at day 3, 7, and 14 after infection with 1×10^4 ^pfu LCMV to analyze the influence of JAM-C overexpression on viral clearance. In LCMV-plaque assays we found the highest viral titer in the pancreas and the spleen at day 3 post-infection, whereas by day 14 after LCMV-infection no virus was detected in all organs analyzed (Figure 8B). Importantly, no significant differences in clearance of LCMV in pancreas and spleen of RIP-LCMV-GP × pHHNS-JAM-C and RIP-LCMV-GP mice was found on days 3, 7 and 14 post LCMV-infection (Figure 8B).

Third, no significant difference in the frequency of LCMV-specific CD8 T cells in the spleen or the PDLN of RIP-LCMV-GP × pHHNS-JAM-C and RIP-LCMV-GP mice was found at day 10 after LCMV-infection (Figure 8C). In both groups of mice the frequency of IFN-γ producing CD8 T cells was approximately 2% in the PDLN and approximately 10% in the spleen upon *ex vivo* stimulation with the immunodominant LCMV peptide GP_33_ (Figure 8C). Thus, endothelial cell overexpression of JAM-C seems to have no influence on the viral clearance, the expansion of β-cell-specific T cells, and the cellular infiltration of the islets in the RIP-LCMV model. Subsequently, no difference in T1D incidence and onset was observed.

## Discussion

It has been shown that blockade of JAM-C, which plays an important role in the transmigration of leukocytes through the endothelial cell layer, reduces cellular infiltration and acinar cell necrosis in cerulein-induced pancreatitis [9]. The goal of the present study was to assess the role of JAM-C during the infiltration of the islets of Langerhans by autoaggressive T cells as occurring during T1D in a virus-induced mouse model. We found that JAM-C expression is upregulated around the islets of Langerhans after infection with LCMV. In particular the highest JAM-C expression was found at the time when most lymphocytes are infiltrating the islets. Indeed, upon blockade of JAM-C with a neutralizing anti-JAM-C antibody we detected a tendency for a reduced T1D incidence in RIP-LCMV-NP and a reversion of some diabetic RIP-LCMV-GP mice. However, we were not able to find significant differences in the degree of cellular infiltration and the frequency of LCMV-specific T cells.

The RIP-LCMV model comes in two flavors, fast onset (RIP-LCMV-GP) and slow onset (RIP-LCMV-NP). Because LCMV-NP is also expressed in the thymus high affinity LCMV-NP-specific T cells are deleted by central tolerance mechanisms [12]. Thus, in contrast to RIP-LCMV-GP mice, LCMV-infected RIP-LCMV-NP mice develop T1D much slower and the autodestructive process is dependent on the presence of LCMV-NP specific CD4 T cells [11]. Due its milder manifestation therapy of T1D was often more successful in the RIP-LCMV-NP than the RIP-LCMV-GP model [15]. In the present study we observed a mild effect on the progression of T1D upon neutralization of JAM-C in the fast-onset RIP-LCMV-GP, but not the slow-onset RIP-LCMV-NP model. However, we did neither find altered frequencies of LCMV-specific T cells in the spleen and the PDLN nor significantly reduced cellular infiltration into the islets of Langerhans.

The lack of a reduction of insulitis was rather surprising, since JAM-C expression was upregulated around the islets of Langerhans and throughout the pancreas upon LCMV-infection and the highest JAM-C expression coincided with the burst of cellular infiltration of the pancreas. It has been previously demonstrated that blockade of JAM-C with the identical neutralizing monoclonal antibody that inhibits both JAM-C homophilic interactions as well as JAM-C/JAM-B heterophilic interactions [16] reduced the accumulation of leukocytes at the site of inflammation [9], [17], [18]. In contrast, it has also been shown that neutrophils adhere and transmigrate on cultured HUVECs under flow in a JAM-C independent manner [19]. However, several experimental models of inflammation in mice suggest a substantial influence of JAM-C on leukocyte extravasation [13], [17], [20]. JAM-C, localized in endothelial-cell tight junctions, was shown to be predominantly involved controlling polarized transmigration. Interestingly, blocking of JAM-C did not directly affect monocyte transendothelial migration under flow, but led to increased monocyte and neutrophil reverse transmigration and return to the circulation [21]. This observation raises the possibility that the disruption of the JAM-B/JAM-C interaction might lead to a shift in the leukocyte entry/exit equilibrium at endothelial junctions. Thus, an increase of leukocytes with a reverse-transmigratory behavior might result in the observed tendency of a reversion of T1D in some RIP-LCMV-GP mice after JAM-C blockade without visible differences in overall insulitis. Due to the ongoing inflammation in the islet leukocytes might remain in close proximity to the islets and might get trapped in a transmigratory loop as suggested [22].

In order to visualize such a possible transmigratory loop we transferred splenocytes of P14 TcR-trangenic mice that exclusively generate CD8 T cells specific for the immunodominat LCMV epitope GP_33_ into RIP-LCMV-GP mice challenged with GP_33_ and Poly(I:C). In this model JAM-C blockade did not diminish CD8^+^ T cell extravasation *in vivo* as analyzed by two-photon microscopy at day 7 to 9 after adoptive transfer of P14 splenocytes (data not shown). Similar to previous experiments [23], [24] visualization of the interaction of infiltrating cells and β-cells within islets demonstrated a highly diverse migration behavior of individual cells. However, no difference between anti-JAM-C antibody-treated and control animals could be observed.

JAM-C overexpression on mouse endothelial cells has been shown to increase the early influx of inflammatory cells into lungs after intraperitoneal LPS challenge [13] and upregulate leukocyte infiltrate and tissue damage in cerulein-induced pancreatitis [9]. In contrast, in our mouse model for T1D JAM-C overexpression had no influence on the development of disease. Early infiltration, viral clearance as well as presence of antigen-specific T cells were similar in double transgenic JAM-C × RIP-LCMV and in single transgenic RIP-LCMV mice. The observed gender difference in the T1D incidence in LCMV-infected RIP-LCMV-NP × pHHNS-JAM-C mice was similar to earlier observations made in the single transgenic RIP-LCMV-NP mouse line. It has become evident in the last 5–10 years that young (6–24 wks of age) male RIP-LCMV-NP mice display an accelerated development of T1D compared to earlier observations [10], [12]. In contrast, male RIP-LCMV-NP mice older than 24 wks of age rarely develop T1D after LCMV-infection (Christen & von Herrath, unpublished observations). No changes to earlier observations have been detected for female RIP-LCMV-NP mice that still display a slow onset T1D starting between 1 to 6 months post-infection.

One reason for the contrary findings in the RIP-LCMV and the cerulein model might be the nature of the different disease model used. In the cerulein-induced pancreatitic model an inflammation within the acinar part of the pancreas was induced by the administration of the chemical compound cerulein that finally resulted in pancreatitis. This seems to depend mainly on the infiltration of neutrophils and monocytes rather than lymphocytes. Hence, the cerulein model is rather different than the virus-induced RIP-LCMV model and its immunopathogenic processes involved in the T cell-mediated destruction of β-cells. An alternative explanation would be that JAM-C overexpression does not have any influence on virus-induced T1D because unlike to antibody treatment JAM-C is overexpressed from the beginning of the development and therefore the immune system might adapt to this exceptional condition. Further, we found that in contrast to cerulein-administration infection with LCMV by itself induces JAM-C upregulation in the pancreas at the time of peak lymphocyte infiltration. Therefore, the additional transgenic overexpression might not have an accelerating effect on the migration of aggressive, islet-specific T cells into the islets. However, other inflammatory factors, such as the chemokine CXCL10, have a dramatic effect on the pathogenesis when overexpressed in the islets of Langerhans [25]. CXCL10 is expressed in the islets upon LCMV infection [26] and its neutralization has been shown to reduce T1D severity in the RIP-LCMV model [1]. Recent evidence however questions a key role for CXCL10 in T1D [27], [28]. Nevertheless, β-cell-specific overexpression of CXCL10 resulted in a spontaneous infiltration of the islets of Langerhans and massively accelerated LCMV-induced T1D in the slow-onset RIP-LCMV-NP model [25].

Thus, JAM-C might only play a minor role in the cellular infiltration of the islets of Langerhans by β-cell specific T cells and subsequently the development of T1D. JAM-C might be more important a factor in the precipitation of lung-associated disorders, such as acute pulmonary inflammation. JAM-C expression is strong in the respiratory tract, which is heavily vascularized and displays high endothelial cell content [13]. Thus, in the lung JAM-C plays a more crucial role in the transmigration of leukocytes through the endothelial cell layer to penetrate the site of inflammation. There is evidence from several studies that adhesion molecules other than JAM-C indeed have an impact on the development of T1D. In an adoptive transfer model of diabetes in NOD mice the neutralization of L-selectin or very late antigen 4 (VLA-4) that are expressed on migrating leukocytes and interact with adhesion molecules expressed on the endothelial cells resulted in a reduction of T1D [29]. Moreover, the treatment with an antibody neutralizing vascular cell adhesion molecule-1 (VCAM-1) delayed the onset of disease, whereas blocking antibody against intercellular adhesion molecule-1 (ICAM-1) had only a marginal effect on the onset of T1D [30]. ICAM-1 is constitutively expressed in low concentrations on endothelial cells and is highly upregulated during inflammation. In contrast, VCAM-1 is only expressed on endothelial cells after cytokine stimulation, binds to VLA-4 and mediates adhesion of leukocytes to the endothelium [31], [32]. These data indicate that the interaction of VLA-4/VCAM-1 seems to be crucial for the rolling and adhesion of leukocytes to the site of injury in this adoptive transfer model of diabetes in NOD mice and the CD11b or lymphocyte function associated antigen 1 (LFA-1) interaction with ICAM-1 has less impact in this mouse model. Other than VCAM-1 and ICAM-1, JAM-C seems to play a minor role in rolling and adhesion of leukocytes and is rather crucial in transmigration of leukocytes through the endothelial cell layer to enter the site of inflammation [20]. JAM-C interacts with CD11b and CD11c mediating extravasation of leukocytes [2], [16]. Collectively these data and our findings in the RIP-LCMV model suggest that JAM-C/CD11b interaction plays only a minor role in the pathogenesis of T1D.
